# PLI-VINS: Visual-Inertial SLAM Based on Point-Line Feature Fusion in Indoor Environment

**DOI:** 10.3390/s22145457

**Published:** 2022-07-21

**Authors:** Zhangzhen Zhao, Tao Song, Bin Xing, Yu Lei, Ziqin Wang

**Affiliations:** 1 Chongqing Key Laboratory of Optical Fiber Sensing and Photoelectric Detection, Chongqing University of Technology, Chongqing 400054, China; bwcxzzz@2020.cqut.edu.cn (Z.Z.); leiyu@2020.cqut.edu.cn (Y.L.); wangziqin1997@stu.cqut.edu.cn (Z.W.); 2 Chongqing Industrial Big Data Innovation Center Co., Ltd., Chongqing 400708, China; xingbin@casic.com.cn

**Keywords:** visual inertial SLAM, indoor environment, point and line feature, nonlinear optimization

## Abstract

In indoor low-texture environments, the point feature-based visual SLAM system has poor robustness and low trajectory accuracy. Therefore, we propose a visual inertial SLAM algorithm based on point-line feature fusion. Firstly, in order to improve the quality of the extracted line segment, a line segment extraction algorithm with adaptive threshold value is proposed. By constructing the adjacent matrix of the line segment and judging the direction of the line segment, it can decide whether to merge or eliminate other line segments. At the same time, geometric constraint line feature matching is considered to improve the efficiency of processing line features. Compared with the traditional algorithm, the processing efficiency of our proposed method is greatly improved. Then, point, line, and inertial data are effectively fused in a sliding window to achieve high-accuracy pose estimation. Finally, experiments on the EuRoC dataset show that the proposed PLI-VINS performs better than the traditional visual inertial SLAM system using point features and point line features.

## 1. Introduction

In recent years, simultaneous localization and mapping (SLAM) has developed into a research hotspot in the field of mobile robots. It is considered to be the core link to achieve autonomous navigation. SLAM includes two main tasks, namely positioning and mapping robots in an unknown environment; the pose of the robot is obtained by detecting the surrounding features of the sensor during the movement process, and then the map of the environment is constructed from the robot’s point of view [1,2].

Visual SLAM can be divided into direct methods and feature methods to estimate camera motion based on the obtained images. Direct methods, such as DTAM [3], LSD-SLAM [4] and DSO [5], estimate camera motion according to the pixel brightness information of the image, and optimize the solution by minimizing the brightness error. However, its prerequisite is based on the assumption of the invariant gray level, that is, the pixel gray level of the same spatial point is fixed in successive image frames. The point feature method mainly uses SIFT [6], ORB [7] or SURF [8] to extract and match point features. According to the result of feature matching, incremental beam adjustment is used to minimize the reprojection error to estimate the camera pose, such as PTAM [9] and ORB-SLAM2 [10].

However, the shortcomings of pure visual SLAM are also obvious. It is more sensitive to situations where the movement speed is too fast, the rotation is too intense, and the exposure is too high. The IMU inertial measurement unit can obtain more accurate motion information because the acquisition frequency is higher than that of the camera, but the IMU also has drift. The effective combination of the two can make up for the shortcomings of the visual degradation of the camera and the drift of the correction IMU, so as to provide better data information. To solve these problems, researchers combine vision and IMU and propose a number of tightly coupled visual-inertial SLAM methods that jointly optimize vision and IMU information, such as MSCKF [11], VINS-Mono [12] and ORB-SLAM3 [13] and so on. VINS-Mono, proposed by Tong Qin in 2018, is a sliding window estimator based on nonlinear optimization. It closely integrates pre-integrated IMU measurements with visual observation, minimizes the sum of IMU measurements and visual measurement residuals, and obtains the maximum posteriori estimation. By solving nonlinear problems, the attitude and deviation of the IMU can be calculated. Carlos Campos et al. proposed ORB-SLAM3 in 2020. This system integrates IMU on the basis of ORB-SLAM2, which greatly improves its performance. It is one of the most advanced visual-inertial SLAM systems based on the feature point method.

In addition, there are often rich line segment features in indoor and other artificial environments, so many researchers add line features to the data association between images. Lu Y [14] et al. implemented visual odometry for point-line feature fusion based on RGB-D cameras, and proved that point-line features have less motion uncertainty than single features. For the first time, Zuo X [15] adopted the orthogonal representation of the minimum parameterized line in SLAM, and analytically derived the Jacobian matrix of the reprojection error relative to the line parameters, which greatly improved the SLAM solution. Gomez O R et al. proposed the PL-SLAM [16] method based on a binocular camera, which uses point-line features in all processes, including visual odometer, key frame selection, and beam adjustment, and proposed a new word bag model, which comprehensively considers the information of point-line features in the closed-loop process. Yi Jia He et al. proposed the PL-VIO [17] method based on the VINS-Mono method, which optimized the point-line features and IMU information in a tightly coupled manner, superior to VIO systems based only on point-features. Li X et al. [18] added surface features and coplanar constraints on the basis of PL-VIO to further model the environmental topology based on the 3D grid generated by point features. However, because the LSD [19] algorithm takes a long time to extract line features, it is difficult to run the algorithms combined with point and line features above in real time. Qiang F et al. proposed PL-VINS [20], which adjusted the implicit parameters of the LSD algorithm and realized real-time operation of the LSD algorithm without affecting accuracy as much as possible. Lee J et al. proposed another VIO system PLF-VINS [21] that integrates point-line features in 2021. It introduces two methods of fusing point and line features. Firstly, the similarity of the relative positions of points and lines is calculated, and then the relationship between points and line features is judged by the position relation, and the parallel line relation is judged in this process.

However, many SLAM systems based on multi-source information fusion still face many challenges in indoor environments. First of all, most point-line based visual SLAM systems do not make full use of line segment information and effectively combine with IMU. Second, the VIO system was unable to extract enough point features from an area with repeated textures. Although many of the above methods are committed to solving these challenges, methods such as [16,17,20] do not fully consider the influence of different types of line segments on camera pose estimation in indoor structural environments.

In order to solve the above problems, we propose a visual-inertial SLAM system based on point-line feature fusion for indoor environments. The main contributions are as follows:

- In order to effectively obtain the structural information of indoor environments and process the environment with repeated texture, an adaptive threshold line segment extraction algorithm is proposed on the premise of point-line feature fusion, which is used to process various redundant line segments in indoor environments to improve the quality of line features.
- The point feature, line feature and IMU information are effectively fused in an optimization-based sliding window to achieve high precision pose estimation. Experiments on EuRoC datasets [22] show that the algorithm presented in this paper has better performance than optical flow-based VINS-Mono and point-line based PL-VINS.

In the remainder of this article, the architecture of the proposed approach is described in Section 2. Section 3 and Section 4 describe in detail the work of the line segment extraction algorithm proposed in this paper in indoor environments and the effective utilization of point and line features and IMU in sliding windows. Section 5 describes the experimental setup and the experimental results under a common data set. Finally, Section 6 provides concluding observations and describes future work.

## 2. System Overview

**Figure 1:**
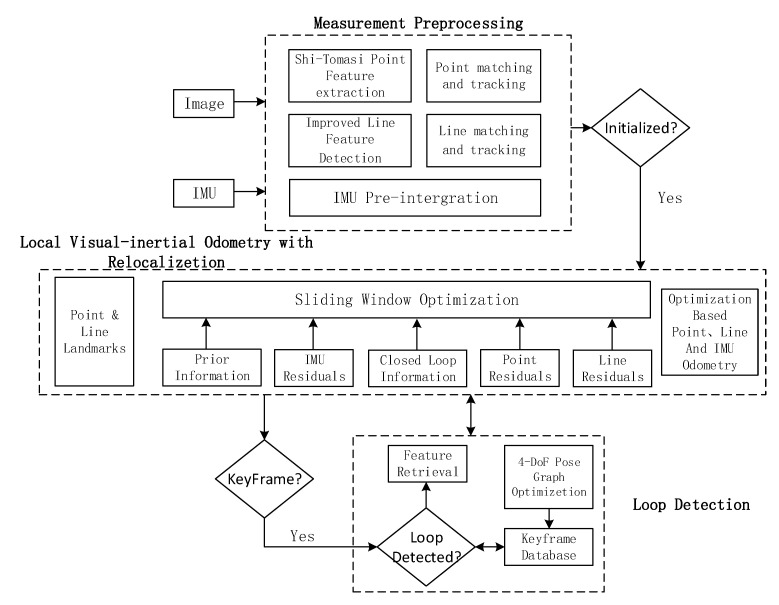
The system of PLI-VINS.

The method proposed in this paper is mainly improved based on the VINS-Mono system. The designed system block diagram is shown in Figure 1, which is mainly divided into measurement preprocessing, local sliding window optimization and closed-loop detection. The initialization process adopts the same strategy as that of VINS-Mono. Based on the loose coupling strategy of visual information IMU information, the pose of all frames in the sliding window and the inverse depth of 3D points are estimated by using the pure visual structure from motion (SFM), and finally aligned with the result of IMU pre-integration. The initialization parameters are solved.

For feature extraction and tracking, one must firstly extract the Shi-Tomasi [23] feature points from the input images, and then match and track feature points based on the optical flow method. For line features, the proposed adaptive threshold line segment extraction algorithm is used to extract line segments, LBD [24] descriptors are calculated, and the KNN [25] algorithm is used to match the distance between descriptor and line segment angle. This process is described in detail in Section 3 of this article.

After system initialization, the point-line feature results are sent to the sliding window optimization section, and the sliding window optimization will pre-integrate IMU data. The nonlinear estimator based on the sliding window model can construct the joint optimization function according to the point-line constraints, IMU constraints and loopback constraints, and solve the position, velocity, rotation and bias of all frames in the sliding window. The detailed contents will be introduced in the third and fourth chapters of this paper.

In the loop detection part, we follow the strategy of VINS-Mono. Firstly, whether to insert key frames is determined according to the parallax between the two frames. If a key frame is inserted, loop detection is performed through the DBoW [26] word bag model and BRIEF [27] descriptor. If there is a loopback, the relocation process is used to maintain alignment between the current the sliding window and the poses map of the past time, and all the poses of the loopback is taken as a constant, and all the IMU measurements, local visual measurements and corresponding feature values extracted from the loopback are used to optimize the sliding window, so as to reduce the cumulative error and calculation amount of the system. However, visual inertia information can provide roll angle and pitch angle data, so there are only four degrees-of-freedom (DOF) errors (triaxial position error and heading angle error). The consistency of the global trajectory can be guaranteed only by adding key frames to the bitmap and optimizing its 4DOF.

## 3. Point Line Feature Processing

**Figure 2:**
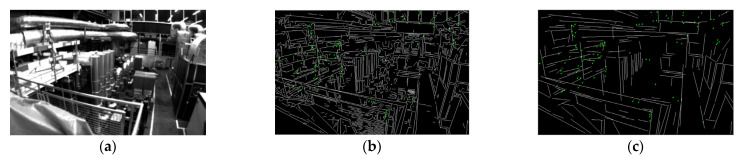
Comparison of the effects of the two algorithms: (**a**) EuRoC datasets scene; (**b**) traditional LSD + KLT optical flow; (**c**) the effect of our algorithm.

For point features in indoor environments, the Shi-Tomasi algorithm is used to detect corner points in this paper, and then the KLT optical flow algorithm [28] is used to track and match feature points, and RANSAC-based pair geometric constraints [29] are used to identify internal and external points and eliminate outliers. For line features in indoor scenes, an adaptive threshold line segment extraction algorithm is proposed to process line features. Subsequently, LBD and KNN were used to describe and match the line features, and the existing line feature outliers were identified by matching the Hamming distance and angle of the line segment. Figure 2 shows the comparison between the traditional LSD and KLT optical flow and the proposed algorithm in the EuRoC datasets factory scenario.

### 3.1. Adaptive Threshold Line Segment Extraction Algorithm

**Figure 3:**
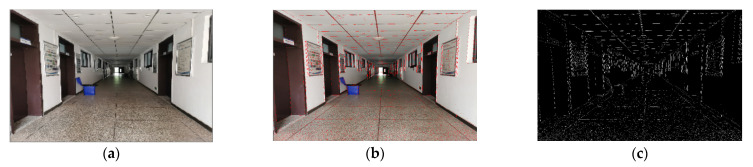
LSD line segment extraction effect: (**a**) indoor promenade scene; (**b**,**c**): LSD line segment extraction effect.

When the traditional LSD algorithm is used in structural scenes, it is easy to produce many short, overlapping and overlapping line segments. As shown in Figure 3b,c, these line segments easily cause matching difficulties, resulting in the decrease in the rate and accuracy of camera pose estimation. We propose an adaptive threshold line segment extraction algorithm, which merges and removes the above-mentioned line segments to further reduce redundant matching and mismatching of line features, thus improving the robustness and accuracy of the proposed algorithm.

Firstly, length screening was carried out for the set $\left\{l_{1},l_{2},\cdots l_{N}\right\}$ of all line segments extracted by the traditional LSD algorithm; the short line segment whose $len_{l_{i}}$ is less than the length threshold $len_{\min}$ is eliminated. The short lines that have great influence on attitude estimation can be deleted by length screening. The length threshold $len_{\min}$ satisfies the following formula:

(1) $$\begin{array}{l} len_{l_{i}}\geq len_{\min},i\subseteq \left\{1,2,\cdots ,N\right\}\\ len_{\min}=\frac{N}{3N+\left\lceil \max(W_{k},H_{k})\right\rceil }\cdot \left\lceil \min(W_{k},H_{k})\right\rceil \end{array}$$

where N is the number of line features extracted from the image of frame K; $W_{k}$ and $H_{k}$ are the width and height of the current k frame; ⌈•⌉ means round up.

**Figure 4:**
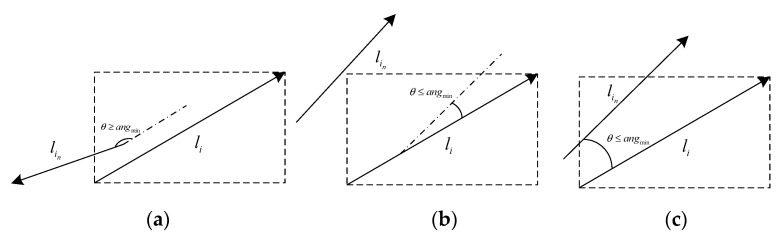
Various line segments: (**a**) the main directions of adjacent line segments are inconsistent; (**b**) adjacent line segments are not in the peripheral matrix of the main line segment; (**c**) set of line segments that meet the conditions for merging.

In the case of the three common line segments as shown in Figure 4, this paper constructs the external matrix of the line segment $l_{i}$ after length screening, and determines whether there are heads, tails and midpoint endpoints of other adjacent line segments in the external matrix area. Then, the line segment features that meet the conditions are added to the same set $\left\{l_{i},l_{i_{1}},l_{i_{2}},\cdots l_{i_{n}}\right\}$. In Figure 4b, no endpoint is located in the external matrix to be eliminated. Since each line segment in the set is characterized by a known starting point and ending point, the main direction A=angle of the vector in the image coordinate system can be calculated. As shown in Figure 4a,c, the main directions of line segment $l_{i}$ and other line segments $l_{i_{n}}$ in the set were calculated and the average value was taken as the angle threshold $ang_{\min}$, and then the features of line segments whose angles with line segment $l_{i}$ were greater than the angle threshold $ang_{\min}$ were eliminated. Finally, all line segments that meet the conditions are extracted from the beginning and end and the midpoint and end points, respectively, and the line segment is fitted to the point set by the least square method.

Compared with the single threshold set by experience in the paper [20,30,31,32], the threshold set in this paper is associated with the number of line segments extracted, image size and scene, which can more effectively adapt to the impact of different indoor scene changes.

LBD descriptors were extracted from the filtered line segments for subsequent feature matching. The KNN algorithm is then used for line segment matching. If the matching distance and angle are less than the threshold value, the matching is considered successful.

### 3.2. Triangulation of Space Line Segments

Using homogeneous coordinates to determine a straight line through two points will generate redundant parameters, which will bring additional computational costs in subsequent optimization. Therefore, this paper introduces Plücker coordinates to represent the straight line. The Plücker coordinate is determined by two different points on the line $L_{W}$.

If one sets straight $L_{W}$ two endpoints of homogeneous coordinates of $p_{1}{\left[x_{1},x_{2},x_{3},x_{4}\right]}^{T}$ and $p_{2}{\left[y_{1},y_{2},y_{3},y_{4}\right]}^{T}$, the straight line $L_{W}$ Plücker coordinates are expressed as follows:

(2) $$L_{W}=\left[\begin{array}{c} p_{1}^{\prime }\times p_{2}^{\prime }\\ x_{4}p_{1}^{\prime }-y_{4}p_{2}^{\prime } \end{array}\right]=\left[\begin{array}{l} n_{w}\\ v_{w} \end{array}\right]\in {\mathbb{R}}^{6}$$

where ${\left[•\right]}_{w}$ represents the coordinates of feature points or feature line segments in the world coordinate system; $p_{1}^{\prime }$ and $p_{2}^{\prime }$ are Cartesian coordinate representations of $p_{1}$ and $p_{2}$, respectively; $n_{w}\in {\mathbb{R}}^{{}^{3}}$ is the normal vector of line $L_{W}$; $v_{w}\in {\mathbb{R}}^{{}^{3}}$ is the direction vector of line $L_{W}$.

The relationship between Plücker matrix T and Plücker coordinates can be obtained as follows:

(3) $$T=p_{2}p_{1}^{T}-p_{1}p_{2}^{T}=[\begin{array}{l} n_{w}^{\wedge }\\ -v_{w}^{T} \end{array}\begin{array}{l} v_{w}\\ 0 \end{array}]$$

where $n_{w}^{\wedge }$ is the antisymmetric matrix of $n_{w}$.

If one allows the transformation matrix of line $L_{W}$ from the world coordinate system to the camera coordinate system be $H_{cw}$, then $H_{cw}$ is as follows:

(4) $$H_{cw}=\left[\begin{array}{cc} R_{cw} & t_{cw}^{\wedge }R_{cw}\\ 0 & R_{cw} \end{array}\right]$$

where $R_{cw}$ and $t_{cw}$ represent the rotation matrix and translation vector of line $L_{W}$ transformed from the world coordinate system to the camera coordinate system.

$L_{c}$ is the coordinate of line $L_{W}$ transformed from the world coordinate system to the camera coordinate system in space, so the formula of Plücker coordinate when representing the coordinate change in line $L_{W}$ is as follows:

(5) $$\begin{array}{l} L_{c}=\left[\begin{array}{l} n_{c}\\ v_{c} \end{array}\right]=[\begin{array}{l} R_{cw}\\ 0 \end{array}\begin{array}{l} t_{cw}^{\wedge }R_{cw}\\ R_{cw} \end{array}]\left[\begin{array}{l} n_{w}\\ v_{w} \end{array}\right]=H_{cw}L_{w}\\ L_{w}=\left[\begin{array}{l} n_{w}\\ v_{w} \end{array}\right]=[\begin{array}{l} R_{cw}^{T}\\ 0 \end{array}\begin{array}{c} -R_{cw}^{T}t_{cw}^{\wedge }\\ R_{cw}^{T} \end{array}]\left[\begin{array}{l} n_{c}\\ v_{c} \end{array}\right]=H_{cw}^{-1}L_{c} \end{array}$$

Space line $L_{c}$ projection to the plane of projection equations expressed by $L_{1}$, $L_{1}$ as follows:

(6) $$L_{1}=\kappa n_{c}=\left[\begin{array}{ccc} f_{y} & 0 & 0\\ 0 & f_{x} & 0\\ -f_{y}c_{x} & -f_{x}c_{y} & f_{x}f_{y} \end{array}\right]n_{c}=\left[\begin{array}{l} l_{1}\\ l_{2}\\ l_{3} \end{array}\right]$$

where κ is the projection matrix of line features.

It can be observed from the above that the Plücker coordinate is an expression form of six parameters, and there are excessive parameterization and orthogonal constraints, which will still cause unnecessary calculations in the optimization process. In this regard, Bartoli [33] proposed a four-parameter orthogonal representation to address the above problems, and this work is adopted in this paper.

Through the QR decomposition of the Plücker line coordinate $L_{W}={\left[n_{w}^{T},v_{w}^{T}\right]}^{T}$, its orthogonal representation (U,W)∈so(3)×so(2) can be obtained, where U and W are as follows:

(7) $$\begin{array}{l} U=\left[\begin{array}{ccc} \frac{n_{w}}{\left||n_{w}|\right|} & \frac{v_{w}}{\left||v_{w}|\right|} & \frac{n_{w}\times v_{w}}{\left||n_{w}\times v_{w}|\right|} \end{array}\right]\in {\mathbb{R}}^{3\times 3}\\ W=\left[\begin{array}{cc} \cos\theta & -\sin\theta \\ \sin\theta & \cos\theta \end{array}\right]=\frac{1}{\sqrt{||n_{w}|{|}^{2}+||v_{w}|{|}^{2}}}\left[\begin{array}{cc} \left||n_{w}|\right| & -||v_{w}||\\ \left||v_{w}|\right| & \left||n_{w}|\right| \end{array}\right]\in {\mathbb{R}}^{2\times 2} \end{array}$$

where U and W represent the three-dimensional and two-dimensional rotation matrices, respectively; θ is the rotation angle.

Then, the Plücker line coordinate $L_{w}^{\prime }$ after orthogonal representation can be expressed as follows:

(8) $$L_{w}^{\prime }={\left[\cos\theta u_{1}^{T},\sin\theta u_{2}^{T}\right]}^{T}$$

where $u_{i}$ represents the ith column of matrix U.

### 3.3. Reprojection Error Model of Line Feature

**Figure 5:**
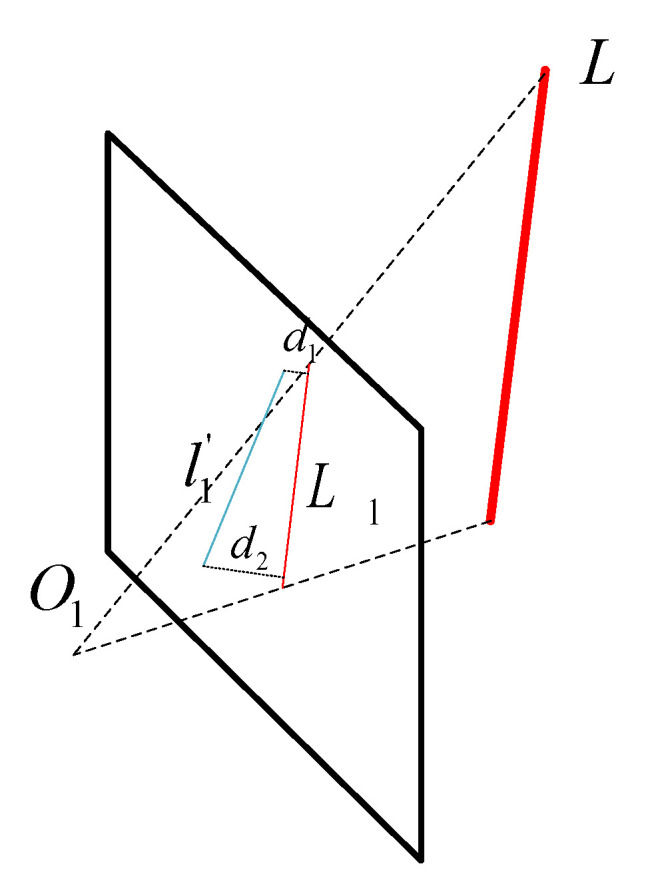
Reprojection error of line features.

As shown in Figure 5, the projection line segments of line L on the image plane are $L_{1}$, and $l_{1}^{\prime }$ is the observation line segment. One must let the end points of $l_{1}^{\prime }$ segment $X_{1}={\left[x_{1},y_{1},1\right]}^{T}$ and $X_{2}={\left[x_{2},y_{2},1\right]}^{T}$, and the projection segment $L_{1}=\left[l_{1},l_{2},l_{3}\right]$.

Then, the distance between the two endpoints and the projected line segment is as follows:

(9) $$e_{l}={\left[\begin{array}{cc} d_{1} & d_{2} \end{array}\right]}^{T}={[\frac{x_{1}^{T}L_{1}}{\sqrt{l_{1}^{2}+l_{2}^{2}}}\frac{x_{2}^{T}L_{1}}{\sqrt{l_{1}^{2}+l_{2}^{2}}}]}^{T}$$

The Jacobian matrix of the camera pose increment can be solved according to the chain rule, which is as follows:

(10) $$J_{L}=\frac{\partial e_{l}}{\partial \delta_{\zeta }}=\frac{\partial e_{l}}{\partial L_{1}}\frac{\partial L_{1}}{\partial L_{c}}\frac{\partial L_{c}}{\partial \delta_{\zeta }}$$

$L_{1}$ and $L_{c}$ can be obtained from Equations (5) and (6), and the three items on the right of Equation (10) are as follows:

(11) $$\begin{array}{l} \frac{\partial e_{l}}{\partial L_{1}}={\left[\begin{array}{ccc} \begin{array}{l} \frac{-l_{1}X_{1}^{T}L_{1}+x_{1}(l_{1}^{2}+l_{2}^{2})}{{\left(l_{1}^{2}+l_{2}^{2}\right)}^{\frac{3}{2}}}\\ \frac{-l_{1}X_{2}^{T}L_{1}+x_{2}(l_{1}^{2}+l_{2}^{2})}{{\left(l_{1}^{2}+l_{2}^{2}\right)}^{\frac{3}{2}}} \end{array} & \begin{array}{l} \frac{-l_{2}X_{1}^{T}L_{1}+y_{1}(l_{1}^{2}+l_{2}^{2})}{{\left(l_{1}^{2}+l_{2}^{2}\right)}^{\frac{3}{2}}}\\ \frac{-l_{2}X_{1}^{T}L_{1}+y_{2}(l_{1}^{2}+l_{2}^{2})}{{\left(l_{1}^{2}+l_{2}^{2}\right)}^{\frac{3}{2}}} \end{array} & \begin{array}{l} \frac{1}{\sqrt{l_{1}^{2}+l_{2}^{2}}}\\ \frac{1}{\sqrt{l_{1}^{2}+l_{2}^{2}}} \end{array} \end{array}\right]}_{2\times 3}\\ \frac{\partial L_{1}}{\partial L_{c}}=\frac{\partial (\kappa n_{c})}{\partial L_{c}}={[\begin{array}{cc} \kappa & 0] \end{array}}_{3\times 6}\\ \frac{\partial L_{c}}{\partial \delta_{\zeta }}={\left[\begin{array}{cc} -{\left(R_{cw}n_{cw}\right)}^{\wedge }-{\left(t_{cw}^{\wedge }R_{cw}v_{w}\right)}^{\wedge } & -{\left(R_{cw}v_{w}\right)}^{\wedge }\\ -{\left(R_{cw}v_{w}\right)}^{\wedge } & 0 \end{array}\right]}_{6\times 6} \end{array}$$

## 4. Nonlinear Optimization Based on Sliding Window

In this paper, the nonlinear optimization method based on the sliding window model is adopted, that is, to ensure that the number of optimization variables is maintained in a certain range, the optimization variables are dynamically added or removed through the sliding window, and only the key frame data in the current period of time participate in the position pose solution process.

The complete state vector at moment *i* in the sliding window is defined as follows:

(12) $$\begin{array}{l} \chi =\left[x_{n},x_{n+1}\cdots x_{n+N},\lambda_{m},\lambda_{m}\cdots \lambda_{m+M},O_{l},O_{l+1}\cdots O_{l+L}\right]\\ x_{i}={\left[p_{\omega b_{i}},q_{\omega b_{i}},v_{\omega b_{i}},b_{b_{i}}^{a},b_{b_{i}}^{g}\right]}^{T},i\in \left[n,n+N\right] \end{array}$$

where $x_{i}$ is IMU state vector at window i, $p_{\omega b_{i}}$ is position information, $q_{\omega b_{i}}$ is pose information, $v_{\omega b_{i}}$ is velocity, $b_{b_{i}}^{a}$ and $b_{b_{i}}^{g}$ are accelerometer bias and gyroscope bias, respectively; $\lambda_{m}$ represents the inverse depth of 3D points; $O_{l}$ is the orthogonal representation of line features in the world coordinate system; N is the number of key frames in the sliding window, m is the number of point features observed by key frames in the sliding window, and l is the number of line features observed by key frames in the sliding window.

On the basis of VINS-Mono, the residual term of line feature is added into the objective optimization function. That is, the objective optimization function includes marginal prior residual, IMU measurement residual, point and line residual. The specific form is as follows:

(13) $$\underset{\chi }{\min}\left\{\begin{array}{l} \rho (||r_{p}-H_{p}\chi |{|}_{\Sigma_{p}}^{2})+\sum_{k\in B}\rho \left(||r_{B}(z_{b_{k+1}}^{b_{k}},\chi )|{|}_{\Sigma_{b_{k}b_{k+1}}}^{2}\right)\\ +\sum_{(i,j)\in D}\rho \left(||r_{D}(z_{j}^{c_{j}},\chi )|{|}_{p_{j}^{c_{j}}}^{2}\right)+\sum_{(i,j)\in l}\rho \left(||r_{l}(z_{L}^{c_{j}},\chi )|{|}_{p_{L}^{c_{j}}}^{2}\right) \end{array}\right\}$$

where B is the IMU measurement data set, D and l are the collection of point features and line features observed at least twice in the image frame, respectively. $||r_{p}-H_{p}\chi |{|}_{\Sigma_{p}}^{2}$ is the marginal prior information, $H_{p}$ is the marginal prior residual Jacobian matrix; $r_{B}(z_{b_{k+1}}^{b_{k}},\chi )$, $r_{D}(z_{j}^{c_{j}},\chi )$ and $r_{l}(z_{L}^{c_{j}},\chi )$ are the residual terms of IMU, point feature and line feature, respectively. ρ(·) is a Cauchy robust function for suppressing outliers.

## 5. Results

To verify the effectiveness of the proposed visual inertial SLAM algorithm based on the fusion point and line features in indoor environments, experiments were carried out using EuRoC datasets. The dataset was collected by a micro aerial vehicle (UAV) at two different scales, industrial factory and indoor room. There are 11 sequences, including binocular stereo (752*480) images, 200 Hz synchronous IMU information, trajectory truth, and calibration files for external and internal parameters of different sensors. These sequences are classified into different levels based on lighting, texture, dynamic motion, or motion blur.

The experimental platform was configured as Intel I7-7700HQ (8 cores @ 2.8ghz) CPU, 16GB memory, no GPU acceleration, and 64-bit Ubuntu 18 operating system.

Firstly, this paper verifies the effectiveness of the proposed improved LSD algorithm in screening invalid line segments in indoor environments, especially in the efficiency of line segment extraction and matching. Then, the root mean square error (RMSE) of absolute trajectory error (ATE) is used to evaluate the effect of the improved LSD algorithm on improving the accuracy of camera pose tracking, and the effect of the nonlinear optimization algorithm with point and line residuals on the accuracy of camera motion trajectory.

### 5.1. Evaluation of Line Feature Extraction Algorithm

In this section, datasets numbered "MH_01_easy" and "MH_03_medium" are selected from the industrial factory environment. In the indoor room environment, select datasets V1_01_easy, V1_03_difficult, and V2_01_easy. Then ten groups of adjacent images were randomly selected from the above datasets for line feature extraction experiment.

**Figure 6:**
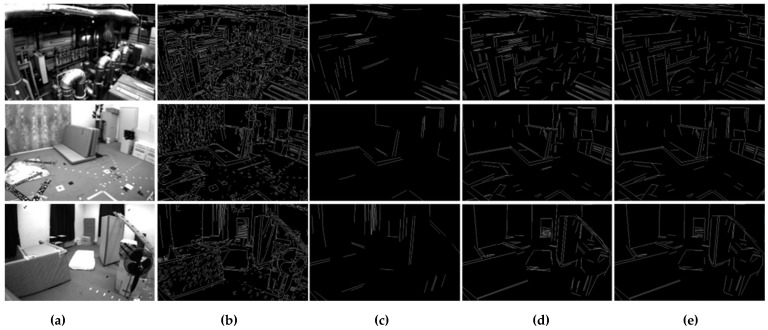
Graph of experimental results: (**a**) three scenarios of EuRoC datasets; (**b**) traditional LSD algorithm; (**c**) LSD algorithm with fixed threshold (line feature extraction algorithm in PL-VINS); (**d**) ours (length filtering); (**e**) ours (length filtering and line segment merging).

Figure 6 (a) shows the scenario of “MH_01_easy”, “V1_01_easy” and “V2_01_easy”; (b) is the line segment graph extracted by the traditional LSD algorithm, in which there are a large number of short, crossed and overlapping line segments. In the calculation of camera pose and position, a large number of repeated and invalid line segments occupy the computing resources. Figure 6c shows the fixed threshold method (line segment length > 60) adopted in PL-VINS. Compared with traditional LSD, it removes the most useless small line segments. The comparison of Figure 6c–e shows that the method adopted by PL-VINS also removes a large number of useful structural line segment features. As shown in Table 1, compared with the traditional LSD and PL-VINS methods, the extraction quantity of the PLI-VINS decreased significantly, and the average running time decreased by 58.5% and 25.6%.

By combining the data in Table 1 and the effect of Figure 6, it can be observed that many unstable short line segments can be screened out by the length factor, and then the adjacent, overlapping, and other line segments that repeatedly describe the same geometric feature type are merged through line segment merging. There are great improvements in efficient line segment representation in indoor scenes and in reducing algorithm running time.

### 5.2. Accuracy Evaluation of Pose Trajectories

**Figure 7:**
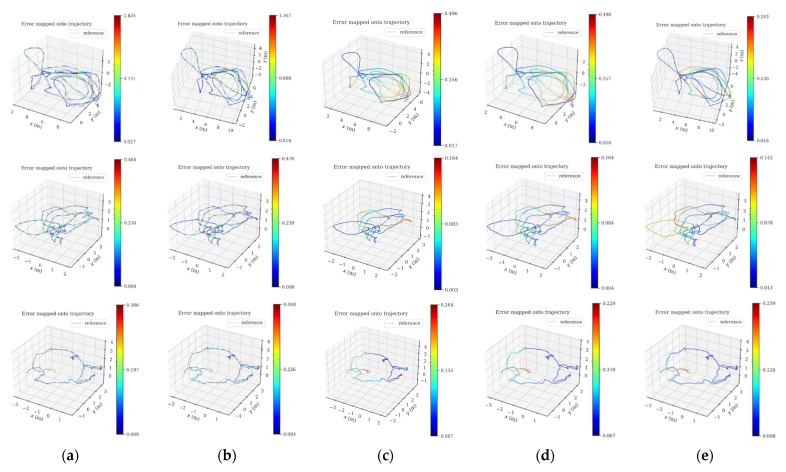
Pose error trajectory comparison of VINS-Mono and ours W/and W/O loop algorithms under sequence MH_03_medium, V1_01_easy and V2_01_easy. The color of the track changes from blue to red, and the closer the color is to red, the greater the error. The gray dotted line is the trajectory truth: (**a**) VINS-Mono w/o loop; (**b**) VINS-Mono; (**c**) ours (length gilter) w/o loop; (**d**) ours (length filter and line merge) w/o loop; (**e**) ours.

In this subsection, the positioning accuracy analysis is performed on all sequences in the EuRoC datasets, and the PLI-VINS is compared with VINS-Mono, PL-VINS and PL-VIO, respectively. The absolute trajectory errors of different algorithms under the EuRoC datasets are shown in Table 2, where the values with the lowest errors are in bold. In Figure 7, this paper shows the accuracy heatmap of VINS-Mono and our algorithm in the sequence MH_03_medium, V1_01_easy, V2_01_easy; the gray dotted line represents the true value of the trajectory, and the colored solid line represents the estimated trajectory. The color of the trace changes from blue to red, indicating a gradual increase in the error of the ATE. Each line shows the results of five methods in the same data set, and the first two of each line are the trajectory of VINS-Mono with no loopback and with loopback. The third is the method track of length filtering only in this paper (no loopback), the fourth is the complete method track of this paper (no loopback), and the last is the track of our algorithm with loopback.

By comparing the three groups of tracks in Figure 7, it can be observed that the proposed method shows better accuracy and stability in the area where the camera has a large rotation. At the same time, compared with the trajectory of VINS-Mono, the trajectory accuracy of the proposed PLI-VINS in V2_01_easy is improved by 40.9%, then it is improved by 53.7% in V1_01_easy, and finally reaches the highest 63.3% in MH_03_medium. The corresponding increases in PL-VINS were only 32.2%, 61.2% and 48.6%. At the same time, by observing the third and fourth track graphs of each group, it can be found that the proposed method performs well in different indoor environments. By properly merging the adjacent line segments to improve the quality of line segments again, the trajectory accuracy of the camera can be effectively improved. Combined with Table 1 and Figure 6 and the operation of the PLI-VINS in the three scenarios, it is not difficult to find that although V1_01_easy and V2_01_easy are indoor scenarios with relatively single environments and limited ability to describe the structural features of line features, this paper improves the quality of line segments by eliminating redundant line segments and merging lines segments; still achieved good trajectory accuracy. However, the MH_03_medium factory scene has a large number of good structural line segment features, which is very conducive to the PLI-VINS to improve the camera trajectory accuracy by using line features. It also shows that the proposed PLI-VINS performs well in various indoor environments.

**Figure 8:**
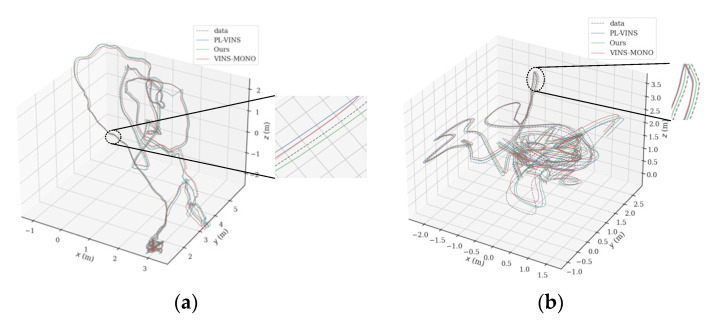
Comparison of trajectories of VINS-Mono, PL-VINS, and PLI-VINS in industrial factories and indoor rooms. The gray dashed lines are the trajectory truth value, and the blue, green and red solid lines are PL-VINS, the proposed PLI-VINS and the VINS-Mono: (**a**) sequence MH_01_easy scene respectively; (**b**) sequence V1_03_difficult scenario.

In terms of root mean square error of absolute trajectory error, as shown in Table 2, the proposed method performs better in almost all EuRoC datasets scenarios. Figure 8 shows the trajectory comparison of the three algorithms in the industrial factory scene of sequence MH_01_easy and the indoor room scene of sequence V1_03_difficult. Compared with PL-VINS, the trajectory accuracy in all scenarios of EuRoC datasets in this paper has smaller errors, especially in difficult scenarios. In all easy scenarios, the RMSE of the proposed PLI-VINS is 0.083, PL-VINS is 0.107, and VINS-Mono is 0.155, respectively, and the trajectory accuracy is improved by 46.5% and 30.9%. However, in difficult scenarios, the trajectory accuracy of the proposed PLI-VINS is improved by 41.3%, while PL-VINS is only 21.1%. It is not difficult to find by referring to the trajectory comparison diagram in Figure 8a,b that in difficult type scenes, the trajectory accuracy of the PLI-VINS is improved more.

## 6. Conclusions

In this paper, a visual-inertial SLAM algorithm based on point-line feature fusion for various indoor environments is proposed. Compared with the visual inertial SLAM algorithm based on point features, the proposed PLI-VINS uses the combination of point and line features to increase the robustness of the visual inertial SLAM system. This PLI-VINS is built on VINS-Mono and evaluated using EuRoC datasets. Different from the existing work, the PLI-VINS makes use of the advantages of different features and sensors, and effectively integrates point, line and IMU data by improving the quality of the extracted line features, thus improving the robustness and accuracy of the system. A comparison with the existing similar work shows that this paper can achieve the highest accuracy in most indoor situations.

In the future, this paper will improve the system by looking for more methods to constrain between 3D lines, and introduce line features into the initialization process, or effectively add line features into the word bag model and dense map of point and line features. These works will further improve the system, will be more suitable for indoor environments, improve the accuracy of camera motion trajectory estimation and the stability of system operation.

## Figures and Tables

**Table 1:** Average line feature extraction quantity and time for different algorithms.

Seq	LSD + LBD + KNN		LSD (Fixed Threshold) + LBD + KNN		Ours + LBD + KNN		
					Length Filter	Length Filter and Line Merge	
	Num	Time (s)	Num	Time (s)	Num	Num	Time (s)
MH_01_easy	2480	5.93	838	3.78	234	179	3.4
MH_03_medium	2328	6.12	1135	3.58	157	132	2.13
V1_01_easy	1562	9.87	763	2.07	128	109	3.48
V1_03_difficult	1757	4.17	957	2.33	78	45	0.74
V2_01_easy	1249	3.3	868	3.3	117	96	1.44
Mean	1875	5.88	912.2	3.01	142.8	112	2.24

**Table 2:** RMSE ATE (M) comparison of VINS-Mono, PL-VINS, PL-VIO and ours (PLI-VINS).

Seq	w/o Loop					w/Loop		
	VINS-Mono	PL-VINS	PL-VIO	Ours (Length Filter)	Ours (Length Filter and Line Merge)	VINS-Mono	PL-VINS	Ours
MH_01_easy	0.244	0.172	0.152	0.232	**0.146**	0.183	0.137	**0.108**
MH_02_easy	0.222	0.193	0.173	0.163	**0.142**	0.175	0.159	**0.090**
MH_03_medium	0.307	0.255	0.265	0.222	**0.214**	0.265	0.136	**0.097**
MH_04_difficult	0.373	0.299	0.363	**0.216**	**0.216**	0.305	0.240	**0.174**
MH_05_difficult	0.421	0.384	0.277	0.255	**0.253**	0.346	0.369	**0.222**
V1_01_easy	0.161	**0.069**	0.098	0.075	0.074	0.145	**0.056**	0.067
V1_02_medium	0.110	0.140	--	**0.123**	**0.123**	0.108	0.097	**0.088**
V1_03_difficult	0.325	0.180	0.201	0.183	**0.148**	0.219	0.162	**0.141**
V2_01_easy	0.135	0.097	0.092	0.103	**0.077**	0.115	0.078	**0.068**
V2_02_medium	0.328	**0.112**	0.155	0.147	0.133	0.259	**0.089**	0.119
V2_03_difficult	0.370	0.214	0.294	**0.136**	0.188	0.303	0.160	**0.152**
Mean	0.272	0.192	0.210	0.169	**0.156**	0.220	0.153	**0.120**

## Data Availability

Not applicable.
